# 2081. COVID-19 mRNA Vaccine Effectiveness against Hospitalizations among U.S. Children and Adolescents During Omicron Variant Predominance

**DOI:** 10.1093/ofid/ofad500.151

**Published:** 2023-11-27

**Authors:** Laura D Zambrano, Margaret M Newhams, Michael J Wu, Regina Simeone, Amber Orzel, Natasha B Halasa, Katherine E Fleming-Dutra, Angela P Campbell, Adrienne G Randolph

**Affiliations:** Centers for Disease Control and Prevention, Atlanta, GA; Boston Children's Hospital, Boston, Massachusetts; Centers for Disease Control and Prevention, Atlanta, GA; Centers for Disease Control and Prevention, Atlanta, GA; Boston Children's Hospital, Boston, Massachusetts; Vanderbilt University Medical Center, Nashville, Tennessee; Centers for Disease Control and Prevention, Atlanta, GA; CDC, Atlanta, GA; Boston Children's Hospital, Harvard Medical School, Boston, Massachusetts

## Abstract

**Background:**

SARS-CoV-2 Omicron variants have increased vaccine-induced immune evasion and their emergence coincided with waning COVID-19 vaccine effectiveness (VE). The duration of immunity from the primary series and VE of booster doses against pediatric COVID-19 (Omicron)-associated hospitalizations has not been characterized in the United States.

**Methods:**

Using a case-control design, we examined VE against laboratory-confirmed COVID-19-associated hospitalizations, enrolling case-patients hospitalized for COVID-19 and SARS-CoV-2 test-negative controls with COVID-like illness from 31 hospitals in 23 states. VE was estimated through multivariable logistic regression by comparing the odds of antecedent primary series or booster COVID-19 mRNA vaccination within and beyond 60 days prior to hospitalization. Results were analyzed by age group (5–11 and 12–18 years) among patients admitted December 19, 2021–March 30, 2023.

**Results:**

We enrolled 1,242 case-patients (972 [78%] of whom were unvaccinated, 164 [13%] of whom received life support, and 11 of whom died) and 1,309 controls. Among children aged 5-18 years, VE of 2 mRNA doses (complete primary series) against pediatric COVID-19 hospitalization was 64% (95% confidence interval [CI]: 44–76%) at 14-60 days after dose 2 (median time since dose 2, 38 days); VE waned to 38% (95% CI: 23–50%) at ≥60 days (median time since vaccination, 213 days). Within 60 days of a monovalent booster dose, VE was 52% (95% CI: -3–77%); among children aged 5-11 years, VE of a bivalent booster dose was 79% (95% CI: 25-94%). Among case-patients who required life support or died, 122/164 (74%) were unvaccinated and only 3 (2%) had received a bivalent booster dose.

**Figure 1.**
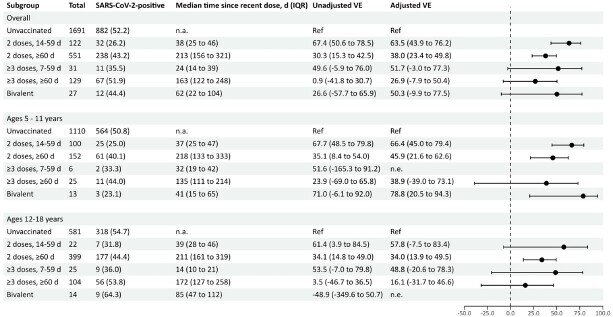
Vaccine effectiveness against COVID-19-related hospitalizations, by age group, vaccine dose, and time since last dose.

VE estimates were only plotted if confidence interval width was <200. Adjusted models included ≥1 underlying medical condition (yes/no), age in years, census region, biweekly date of hospital admission, and social vulnerability index (SVI) score. Sex and race/ethnicity were tested as confounders in the full model but were dropped for absence of evidence of confounding.

**Table 1.**
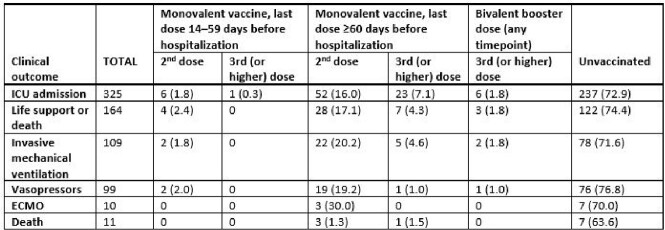
Critical outcomes among enrolled COVID-19 case-patients ages 5-18 years, by vaccine dose and time since last vaccine dose.

Abbreviations: ICU, intensive care unit; ECMO, extracorporeal membrane oxygenation

**Conclusion:**

COVID-19 mRNA vaccination reduced the likelihood of pediatric COVID-19 hospitalization by >60%; VE waned but remained protective, even at a median of 213 days after dose 2. Although not reaching statistical significance due to limited power, monovalent boosters appeared to temporarily restore protection, and bivalent booster doses among 5–11-year-olds were nearly 80% protective against hospitalization. Nearly three quarters of children requiring life support or who died were unvaccinated.

**Disclosures:**

**Regina Simeone, PhD**, Pfizer: Stocks/Bonds **Natasha B. Halasa, MD, MPH**, Merck: Grant/Research Support|Quidell: Grant/Research Support|Quidell: donation of kits|Sanofi: Grant/Research Support|Sanofi: vaccine support

